# METTL3-Mediated LINC00475 Alternative Splicing Promotes Glioma Progression by Inducing Mitochondrial Fission

**DOI:** 10.34133/research.0324

**Published:** 2024-02-23

**Authors:** Yaping Yan, Ailing Luo, Shanshan Liu, Mansi Cai, Xiaodan Liu, Xiaohong Zhang, Siyi Zhang, Yu Liu, Jiamin Zeng, Xinke Xu, Na Zhang, Zhuorong Zhang, Yingyi Xu, Jing He, Xiaoping Liu

**Affiliations:** ^1^ Department of Hematology and Oncology, Guangzhou Women and Children’s Medical Center, Guangzhou Medical University, Guangdong Provincial Clinical Research Center for Child Health, Guangzhou 510623, China.; ^2^ Division of Birth Cohort Study, Guangzhou Women and Children’s Medical Center, Guangzhou Medical University, Guangdong Provincial Clinical Research Center for Child Health, Guangzhou 510623, China.; ^3^ Department of Anesthesiology, Guangzhou Women and Children’s Medical Center, Guangzhou Medical University, Guangdong Provincial Clinical Research Center for Child Health, Guangzhou 510623, China.; ^4^ Department of Nephrology, Center of Kidney and Urology, The Seventh Affiliated Hospital, Sun Yat-sen University, Shenzhen 518107, China.; ^5^ Department of Anesthesiology, The Second Affiliated Hospital of University of South China, Hengyang, Hunan Province 421001, China.; ^6^ Department of Neurosurgery, Guangzhou Women and Children’s Medical Center, Guangzhou Medical University, Guangdong Provincial Clinical Research Center for Child Health, Guangzhou 510623, China.; ^7^ Department of Pediatric Surgery, Guangzhou Women and Children’s Medical Center, Guangzhou Medical University, Guangdong Provincial Clinical Research Center for Child Health, Guangzhou 510623, China.; ^8^ Department of Pediatric Surgery, Guangzhou Institute of Pediatrics, Guangdong Provincial Key Laboratory of Research in Structural Birth Defect Disease, Guangzhou Women and Children’s Medical Center, Guangzhou Medical University, Guangzhou 510623, China.

## Abstract

Mitochondrial fission promotes glioma progression. The function and regulation mechanisms of lncRNAs in glioma mitochondrial fission are unclear. The expression of LINC00475 and its correlation with clinical parameters in glioma were analyzed using bioinformatics. Then, in vitro and in vivo assays were performed to explore the function of spliced variant LINC00475 (LINC00475-S) in gliomas. To explore the mechanisms, RNA-seq, MeRIP, RIP, pulldown-IP, dCas9-ALKBH5 editing system, LC/MS, and Western blotting were utilized. LINC00475 was confirmed to be overexpressed and with higher frequencies of AS events in gliomas compared to normal brain tissue and was associated with worse prognosis. In vitro and animal tumor formation experiments demonstrated that the effect of LINC00475-S on proliferation, metastasis, autophagy, and mitochondrial fission of glioma cells was significantly stronger than that of LINC00475. Mechanistically, METTL3 induced the generation of LINC00475-S by splicing LINC00475 through m6A modification and subsequently promotes mitochondrial fission in glioma cells by inhibiting the expression of MIF. Pull-down combined LC/MS and RIP assays identified that the m6A recognition protein HNRNPH1 bound to LINC00475 within GYR and GY domains and promoted LINC00475 splicing. METTL3 facilitated HNRNPH1 binding to LINC00475 in an m6A-dependent manner, thereby inducing generation of LINC00475-S. METTL3 facilitated HNRNPH1-mediated AS of LINC00475, which promoted glioma progression by inducing mitochondrial fission. Targeting AS of LINC00475 and m6A editing could serve as a therapeutic strategy against gliomas.

## Introduction

Glioma stands as the most prevalent central nervous system tumor [1]. Current treatment approaches for glioma primarily focus on integrated management involving surgery in combination with radiotherapy and chemotherapy, while targeted therapies and biological therapies are also included as complementary measures; to date, these efforts have improved overall survival for low-grade gliomas (LGGs), but not remarkably improved for glioblastoma (GBM) [2].

Mitochondrial fusion and fission exhibit pivotal roles in maintaining mitochondrial homeostasis and function. Mitochondrial fusion facilitates elongation of the mitochondrial structure, thereby enhancing adenosine triphosphate (ATP) production, and metabolite transportation between fused mitochondria. On the other hand, mitochondrial fission promotes the formation of new smaller mitochondria, which are resistant to autophagy, and removes dysfunctional mitochondria through mitophagy. DRP1 (dynamin-related protein 1) participates in fission after recruitment by membrane-anchored mitochondrial fission factor and Fis1 [3]. Mitochondrial fusion proteins MFN1/2 (mitofusin 1/2) are implicated in outer mitochondrial membrane fusion, while OPA1 (GTP-enzyme optic atrophy 1) participates in inner mitochondrial membrane fusion [4]. Mitochondrial fission is considered as a promising therapeutic strategy for tumor therapy [5]. Multiple evidences suggest that balancing mitochondrial dynamics enhances chemotherapy sensitivity and facilitates metastasis and progression in GBM [6]. Nevertheless, the underlying mechanisms responsible for the augmented mitochondrial fission in gliomas remain elusive.

Long noncoding RNAs (lncRNAs) are a class of RNAs that lack protein-coding potential and exceed 200 nucleotides (nt) in length [7]. LncRNAs play opposite roles in various biological processes and are involved in tumorigenesis, including gliomas [8]. Given the close association between dysregulated lncRNAs expression and gliomas, they hold promise for therapeutics interventions and prognostic prediction related to gliomas [9]. Although limited reports have displayed the involvement of lncRNAs in regulating mitochondrial fission in tumors, there is currently no evidence regarding the impact on mitochondrial fission in glioma.

Alternative splicing (AS) is a crucial process in the regulation of posttranscriptional gene expression in eukaryotes, wherein RNA precursors generate diverse mature RNA isoforms through distinct splicing patterns. There are seven main types of AS in organisms: (a) exon skipping, (b) intron retention, (c) alternative 5′ splice site, (d) alternative 3′ splice site, (e) alternative first exon, (f) alternative last exon, and (g) mutually exclusive exon [10]. Tissue-specific AS, encompassing both mRNAs and lncRNAs, occurs in over 90% of human genes [11]. AS is an oncogenic initiation mechanism in glioma [12]. Notably, AS of noncoding RNAs, particularly lncRNAs, plays a pivotal role in the progression and prognosis of cancers [13]. In diffuse intrinsic pontine gliomas, the reduced expression of lncRNA XIST and its splice variant XIST-201 is positively correlated with poor prognosis in children [14]. Therefore, the impact and mechanisms of AS on the functional diversity of lncRNA splicing variants in gliomas remain poorly explored.

m6A, the methylation of the sixth nitrogen atom of adenosine, represents the most common modification in mRNAs and lncRNAs. It plays a crucial role in several aspects of RNA biology including splicing, stability, and translation [15]. The dynamic regulation of m6A is orchestrated by a repertoire of enzymes such as methyltransferases (METTL3, METTL14, WTAP, RBM15/15B, and KIAA1429), demethylases (FTO and ALKBH5), as well as recognition proteins (YTH family, IGF2 mRNA binding proteins, and heterogeneous nuclear ribonucleoprotein family) [16]. In GBM cells, dysregulation of multiple m6A modifiers has been implicated in gene splicing that drives tumor development [17]. However, it remains unclear whether m6A-mediated the AS events occurring within lncRNAs contribute to mitochondrial fission in tumorigenesis.

LINC00475 is a 2,000-nt lncRNA, located in chromosome 9q22.31, is up-regulated in gliomas, and is closely related to an unfavorable prognosis [18]. It has an oncogenic role in gliomas [18,19]. Notably, our recent investigation has unveiled the presence of a cleaved variant of LINC00475, which promotes mitochondrial fission in glioma cells. Exon 1 skipping of LINC00475 forms the AS isoform LINC00475-S. In this study, we aimed to elucidate the underlying mechanisms responsible for the AS of LINC00475 and its induction of mitochondrial fission in glioma.

## Results

### Spliced LINC00475 is up-regulated and related with poor prognosis in gliomas

**Fig. 1.**
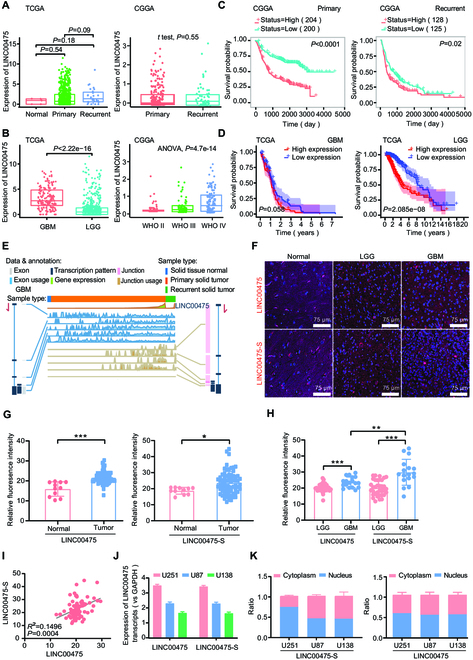
The expression and spliced LINC00475-S was increased in gliomas. (A) The expression of LINC00475 in normal brain tissues and primary and recurrent gliomas was analyzed using the TCGA and CGGA databases. (B) The expression of LINC00475 was examined in different histological grades using the TCGA and CGGA databases. The correlation of LINC00475 expression with prognosis in primary and recurrent glioma patients based on the CGGA dataset (C) and in GBM and LGG based on the TCGA dataset (D). (E) Alternative splicing event analysis of LINC00475 in GBM was conducted using the TSVdb databases. (F) The FISH assay detected the expression and localization of LINC00475-S (red) and LINC00475 (red) in normal brain tissues and GBM and LGG from tissue microarrays. Nuclei were stained by 4′,6-diamidino-2-phenylindole (DAPI) (blue). (G) The fluorescence intensity of LINC00475 (left) and LINC00475-S (right) was calculated in normal brain tissues and gliomas from tissue microarrays. (H) The fluorescence intensity of LINC00475 and LINC00475-S was determined in LGGs and GBMs from tissue microarrays. (I) Pearson’s correlation analysis revealed a correlation between the expressions of LINC00475-S and LINC00475 in gliomas from tissue microarrays. (J) RT-qPCR assays demonstrated relative expressions of LINC00475 and LINC00475-S in glioma U251, U87, and U138 cells. (K) RT-qPCR was performed to determine the presence of LINC00475 and LINC00475-S in nuclear and cytoplasmic fractions of U251, U87, and U138 cell lysates. GAPDH and U6 were used as positive controls. *P < 0.05, **P < 0.01, ***P < 0.001.

Analysis of The Cancer Genome Atlas (TCGA) data revealed a significant up-regulation of LINC00475 expression in recurrent gliomas compared to primary gliomas and normal brain tissues; however, no marked difference was observed between recurrent and primary gliomas from the CGGA data (Fig. 1A). Moreover, LINC00475 expression was higher in GBMs than in LGGs and exhibited an increasing trend with histological grades (Fig. 1B). CGGA data showed elevated LINC00475 expression in both primary and recurrent GBMs compared to normal brain tissues, and no substantial difference between primary and recurrent GBMs; no remarkable difference existed among normal brain tissues, primary LGGs, and recurrent LGGs (Fig. S1A). Similarly, no difference of LINC00475 expression was identified between primary and recurrent patients among different glioma grades (Fig. S1B), nor with gender (Fig. S1C). However, LINC00475 expression was elevated in high-grade gliomas in patients aged 42 years or older compared to their younger counterparts (Fig. S1D). Additionally, isocitrate dehydrogenase 1 (IDH1) wild-type gliomas demonstrated a pronounced LINC00475 overexpression relative to IDH1 mutant gliomas (Fig. S1E), whereas, specifically in World Health Organization (WHO) grade III gliomas, patients without 1p/19q co-deletion exhibited markedly higher levels of LINC00475 relative to those with deletion status (Fig. S1F). It was revealed that patients with high LINC00475 expression displayed a worse prognosis than those with low expression in primary and recurrent gliomas from CGGA database (Fig. 1C). LGG patients with high LINC00475 expression experienced a notably inferior prognosis when compared to individuals with low LINC00475 expression from TCGA database; however, no substantial difference was observed in GBMs (Fig. 1D). Moreover, utilizing the TSVdb online software, a higher frequency of AS in LINC00475 was observed in primary and recurrent GBMs compared to normal brain tissues (Fig. 1E), and the frequency of AS in primary LGGs was higher than in recurrent LGGs (Fig. S1G). To corroborate this discovery, we employed fluorescence in situ hybridization (FISH) to assess the expression of truncated LINC00475 (LINC00475-S, 1,184 nt) and full-length LINC00475 (2,000 nt) in glioma tissues (Fig. 1F and Fig. S1H). The results demonstrated a significantly elevated expression of both forms in glioma tissues compared to normal brain tissues (Fig. 1F), with both forms being up-regulated in GBMs compared to LGGs (Fig. 1G and H and Fig. S1I). Furthermore, a positive correlation between the LINC00475-S expression and full-length LINC00475 in glioma tissues exists (Fig. 1I). The highest level of LINC00475-S was exhibited in U251 cells, followed by U87 cells and U138 cells (Fig. 1J), and LINC00475 was found to be localized in both the cytoplasm and nucleus of glioma cells through nucleocytoplasmic separation experiment (Fig. 1K). Despite the differences in expression of LINC00475 between GBMs and LGGs and its relation to prognosis in different populations, its expression increased significantly in gliomas.

### LINC00475-S promotes mitochondrial fission in glioma cells

**Fig. 2.**
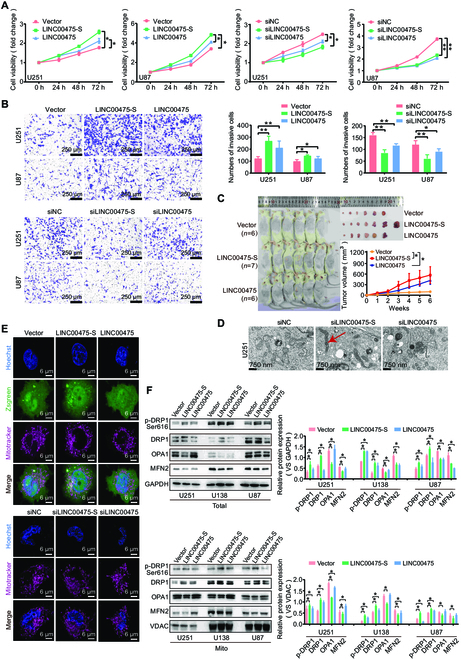
The ability of LINC00475-S promoting glioma progression was stronger than LINC00475. (A) CCK8 assays were employed to assess the viability of U251 and U87 cells transfected with LINC00475-S and LINC00475 overexpression plasmids or siRNA. (B) Transwell assays demonstrated the migratory abilities of U251 and U87 cells transfected with LINC00475-S and LINC00475 overexpression plasmids or siRNA. (C) A xenograft tumor model was established in nude mice by injecting U251 cells transfected with mock vector (*n* = 6), LINC00475-S-overexpressing vector (*n* = 7), and LINC00475-overexpressing vector (*n* = 6), followed by weekly monitoring of tumor volume for 6 weeks. (D) The organelle structures in U251 cells treated with LINC00475 and LINC00475-S siRNA were observed using an electron microscope, where fusion mitochondria were indicated by red arrows. (E) Visualization of mitochondrial morphology in U251 and U87 cells transfected with LINC00475-S and LINC00475 overexpression plasmids. siRNA was performed using a confocal microscope, hoechst33254 for nuclear staining (blue), mitotracker for mitochondria (purple), and zsgreen for vector (green). (F) Western blotting analysis revealed the levels of cellular and mitochondrial DRP1, p-DRP1, OPA1, MFN2, and MIF proteins in LINC00475-S- or LINC00475-overexpressing conditions. GAPDH and VDAC served as internal control for cellular and mitochondrial proteins, respectively. The measurement data were presented as mean ± SD. All tests in this study were repeated three times. *P < 0.05, **P < 0.01.

To investigate the function of LINC00475-S in gliomas, we modulated the expression of LINC00475 and LINC00475-S in glioma cells (Fig. S2A). Cell counting kit-8 (CCK8) assays and Transwell assays demonstrated that overexpression of LINC00475-S led to increasing proliferation and migration capacities of U251 and U87 cells when compared to the LINC00475 group and the control group. Conversely, inhibition of LINC00475-S expression resulted in a notable reduction in proliferation and migration rates compared to the silenced LINC00475 group and the control group (Fig. 2A and B). In vivo experiments suggested an augmented tumorigenic potential upon overexpression LINC00475-S in U251 cells that surpassed what was observed with either LINC00475 overexpression or the control group (Fig. 2C). However, it was important to note that neither LINC00475-S nor LINC00475 exerted significant effect on apoptosis (Fig. S2B). Overexpression of LINC00745-S suppressed autophagy by up-regulating p62 protein while decreasing LC3 II/I ratio. Conversely, inhibition of LINC00475-S stimulated autophagy by down-regulating p62 and increasing LC3 II/I ratio, with a more pronounced effect compared to LINC00475 (Fig. S2C and D). We further investigated alterations in mitochondrial morphology using transmission electron microscopy following modulation of LINC00475-S expression in U251 cells. Surprisingly, we observed elongated and fused mitochondria when inhibiting LINC00475-S expression, whereas only autophagosomes and autolysosomes were evident upon inhibition of LINC00475 expression (Fig. 2D). Overexpression of LINC00475-S led to the presence of punctate mitochondria with significantly reduced length compared to both the control group and LINC00475 group in U251 cells observed by mitotracker staining. Conversely, suppression of LINC00475-S resulted in noticeably elongated mitochondria, surpassing the other two groups (Fig. 2E). Notably, both cytoplasmic and mitochondrial protein analysis demonstrated that overexpression of LINC00475-S enhanced the expression of DRP1 and p-DRP1 while suppressing the expression of OPA1 and MFN2. Importantly, this regulatory effect was stronger than that induced by LINC00475 (Fig. 2F). These results collectively indicated that LINC00475-S could promote glioma cell mitochondrial fission with a significantly greater impact than LINC00475.

### m6A modification facilitates AS of LINC00475 and mitochondria fission in glioma cells

**Fig. 3.**
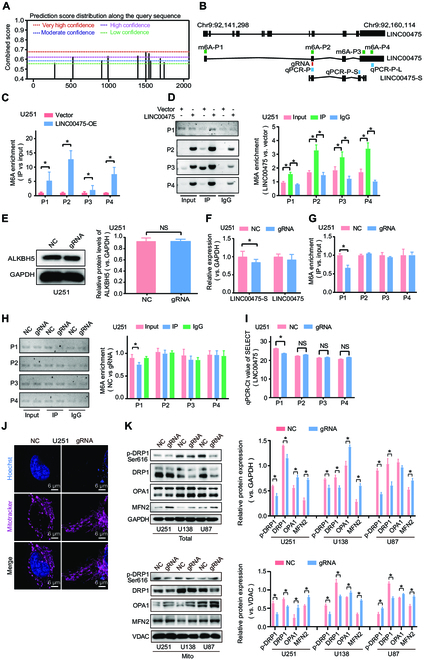
The m6A demethylation of LINC00475 modified by dCas13b-ALKBH5 system. (A) Multiple m6A methylation binding sites within the exon region of LINC00475 predicted through SRAMP database. (B) Schematic diagram illustrated primer design for MeRIP-qPCR and MeRIP-PCR. MeRIP-qPCR (C) assays, along with agarose gel images (D), were utilized to detect m6A enrichment in LINC00475 between vector control group and LINC00475-overexpressed group in U251 cells. (E) ALKBH5 expression following dCas13b-ALKBH5 system-based modification in U25l cells detected by Western blotting. GAPDH was used as internal control. (F) Relative expression of LINC00475-S and LINC00475 after m6A demethylation modification in U251 cells using dCas13b-ALKBH5 and gRNA system using RT-qPCR. The m6A enrichment of LINC00475 in U251 cells was assessed by MeRIP-qPCR (G) and agarose gel images of MeRIP-PCR (H) following m6A demethylation modification. (I) The m6A enrichment at the methylation site of LINC00475 was evaluated by SELECT-qPCR after transfection with dCas13b-ALKBH5 and gRNA plasmids into U25l cells; input was a positive control. (J) Confocal microscopy was employed to visualize the mitochondrial morphology in U251 cells modified with dCas13b-ALKBH5 and gRNA, hoechst33254 for nucleus (blue) and mitotracker for mitochondria (purple). (K) Western blotting was performed to determine the cellular and mitochondrial protein levels of DRP1, p-DRP1, OPA1, MFN2, and MIF following m6A demethylation. GAPDH and VDAC served as positive controls. GAPDH and VDAC served as internal control for cellular and mitochondrial proteins, respectively. The measurement data were presented as mean ± SD. All tests in this study were repeated three times. *P < 0.05. NS, nonsignificant.

Initially, multiple m6A modification sites were predicted in the exons of LINC00475 using the online software m6Avar (Fig. 3A). Subsequently, specific primers were designed based on the sequence characteristics of these sites (Fig. 3B). Methylated RNA immunoprecipitation (MeRIP)–quantitative polymerase chain reaction (qPCR) (Fig. 3C) and MeRIP-PCR gel electrophoresis (Fig. 3D) revealed a significant increase in m6A enrichment on LINC00475 in U251 cells. Given that exon 2 exhibited the highest level of m6A enrichment, we targeted it for demethylation modification (Fig. 3B). After targeted modification of exon 2 using dCas13b-ALKBH5 system (Fig. 3E), there was a significant down-regulation of LINC00475-S in U251 cells, while the expression of LINC00475 remained unchanged (Fig. 3F). The m6A enrichment on exon 1 also significantly decreased, while there was no remarkable change in the m6A enrichment on exons 2 to 4 (Fig. 3G to I). Additionally, mitochondria fusion in U251 cells in the guide RNA (gRNA) group compared to the control group was observed (Fig. 3J). Overall, after targeted modification of exon 2, there was a decrease in cytoplasmic and mitochondrial expression of DRP1 and p-DRP1 as well as an up-regulation of OPA1 and MFN2 expression (Fig. 3K). These findings suggested that m6A demethylation modification on LINC00475 leads to reduced expression of LINC00475-S in U251 cells and promotes increased mitochondrial fusion.

### METTL3 promotes AS of LINC00475 via m6A modification

**Fig. 4.**
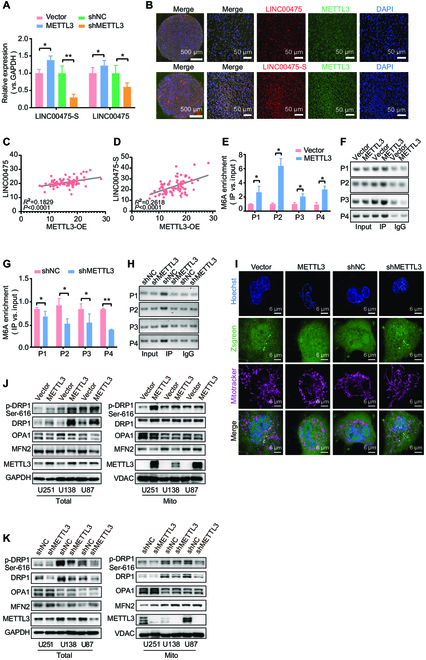
METTL3 facilitated splicing of LINC00475 to induce mitochondrial fission in glioma cells. (A) RT-qPCR was used to assess the expression of LINC00475-S and LINC00475 infected with METTL3 overexpression lentivirus or shRNA in U251 cells. (B) FISH combined with immunofluorescence staining on tissue microarrays demonstrated colocalization of LINC00475-S/LINC00475 (red) and METTL3 (green). Nucleus was stained with DAPI (blue). Pearson’s correlation analysis measured the correlation between LINC00475 (C)/LINC00475-S (D) expression and METTL3 expression in tissue microarrays. MeRIP-qPCR (E) and agarose gel images of MeRIP-PCR (F) were used to evaluate the m6A enrichment of LINC00475 in U251 cells after METTL3 overexpression. MeRIP-qPCR (G) and agarose gel images of MeRIP-PCR (H) determined the m6A enrichment of LINC00475 in U251 cells after METTL3 knockdown. (I) The mitochondrial morphology of U251 cells infected with METTL3 overexpression lentivirus or shRNA visualized using a confocal microscope, hoechst33254 for nuclear staining (blue), mitotracker for mitochondria (purple), zsgreen for vector (green). Western blotting was conducted to detect the cellular and mitochondrial protein levels of DRP1, p-DRP1, OPA1, MFN2, and MIF in METTL3-overexpressed (J) or METTL3-silenced (K) U251 cells. GAPDH and VDAC served as internal control for cellular and mitochondrial proteins, respectively. The measurement data were presented as mean ± SD. All tests in this study were repeated three times. *P < 0.05, **P < 0.01.

To elucidate the role of m6A modifiers in regulating AS of LINC00475, we initially assessed the expression levels of METTL3, ALKBH5, and FTO in glioma cells. Our findings demonstrated that all three proteins were expressed in glioma cells (Fig. S3A). Subsequently, overexpression experiments for METTL3, FTO, and ALKBH5 were conducted in glioma cells (Fig. S3B and C). Quantitative reverse transcription PCR (qRT-PCR) analysis revealed that among the three m6A modification proteins, only overexpression of METTL3 up-regulated both LINC00475 and LINC00475-S, and inhibition of METTL3 led to a significant reduction of their expression. Notably, the regulatory effect exerted by METTL3 on LINC00475-S was stronger than its effect on LINC00475 (Fig. 4A). Furthermore, we observed a significant colocalization between METTL3 and LINC00475-S in glioma tissues (Fig. 4B). The expression of LINC00475 and LINC00475-S was also significantly increased by ALKBH5 and FTO (Fig. S3D). However, the elevation of m6A enrichment in glioma cells up-regulated LINC00475 and the generation of LINC00475-S. Hence, we proposed that METTL3-mediated m6A methylation is implicated in the splicing of LINC00475. A significant up-regulation of METTL3 was observed in glioma compared to normal brain tissues (Fig. S3E), and was significantly higher in GBMs than in LGGs (Fig. S3F). A positive correlation between METTL3 and both LINC00475 (Fig. 4C) and LINC00475-S (Fig. 4D) was identified in glioma tissues. The expression of METTL3 in LGGs exhibited a positive correlation with the levels of LINC00475-S (Fig. S3G), while no marked correlation was observed with LINC00475 (Fig. S3H). In GBMs, no substantial association was demonstrated between METTL3 and LINC00475-S (Fig. S3I), but with LINC00475 (Fig. S3J). The MeRIP analyses demonstrated that the overexpression of METTL3 significantly enhanced m6A enrichment on every exon of LINC00475 (Fig. 4E and F and Fig. S3K). Conversely, inhibition of METTL3 expression led to a reduction of m6A enrichment on LINC00475 (Fig. 4G and H and Fig. S3L). Overexpression of METTL3 promoted mitochondrial fission, whereas its suppression resulted in increased mitochondrial fusion in U251 cells (Fig. 4I). Furthermore, overexpression of METTL3 up-regulated DRP1 and p-DRP1 proteins in both glioma cell cytoplasm and mitochondria while down-regulating OPA1 and MFN2 proteins. On the contrary, restrain of METTL3 suppressed DRP1 and p-DRP1 proteins but up-regulated OPA1 and MFN2 proteins (Fig. 4J and K and Fig. S3M and N). These results suggested that METTL3 induced mitochondrial fission in glioma cells by promoting the formation of LINC00475-S.

### METTL3 and LINC00475-S induce mitochondrial fission by suppressing MIF in glioma cells

**Fig. 5.**
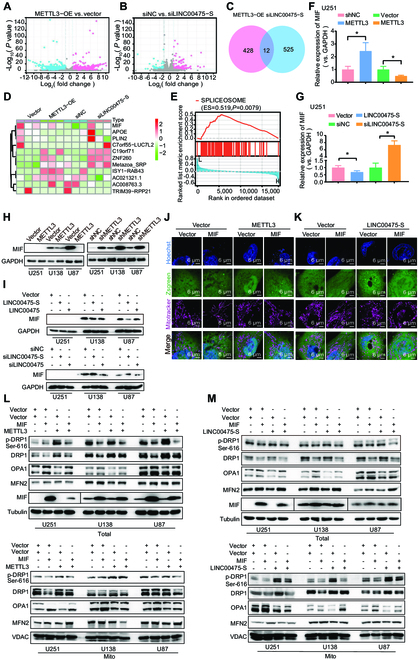
METTL3 and LINC00475-S induced mitochondrial fission by inhibiting MIF. Volcano plot illustrating differentially expressed genes in METTL3-overexpressed (A)/LINC00475-S-silenced (B) U251 cells by RNA-seq analysis. Red dots: up-regulated genes, blue dots: down-regulated genes, gray dots: no substantial differential expression genes (*P* < 0.05, |log2FC| ≥ 1). (C) Venn diagram displayed significantly intersecting targets between METTL3-overexpressed and LINC00475-S knockdown-induced target genes. (D) Heatmap showing the expression of screened 12 mRNAs obtained from intersection from (C). (E) GSEA results demonstrated enrichment of intersection genes in the spliceosome pathway. (F) RT-qPCR was employed to assess relative MIF expression levels in U251 cells infected with METTL3-OE lentivirus or shMETTL3 lentivirus. (G) RT-qPCR was used to evaluate relative MIF expression in U251 cells transfected with LINC00475-S plasmids or LINC00475-S siRNA (siLINC00475-S). The protein level of MIF in U251, U138, and U87 cells transfected with METTL3 overexpression or shRNA lentivirus (H) and LINC00475-S overexpression or knockdown (siLINC00475-S) plasmids (I) was determined using Western blotting. Confocal microscopy was employed to visualize the mitochondrial morphology in METTL3-overexpressed (J) or LINC00475-S-overexpressed (K) U251 cells infected with MIF-overexpressing lentivirus, hoechst33254 for nuclear staining (blue), mitotracker for mitochondria (purple), zsgreen for vector (green). Western blotting analysis was performed to detect the cellular protein (Total) and mitochondrial (Mito) protein levels of DRP1, p-DRP1, OPA1, MFN2, and MIF in METTL3-overexpressed (L) or LINC00475-S-overexpressed (M) U251 cells infected with MIF-overexpressing lentivirus. GAPDH and VDAC served as internal control for cellular and mitochondrial proteins, respectively. The measurement data were presented as mean ± SD. All tests in this study were repeated three times. *P < 0.05.

In order to investigate the mechanism underlying METTL3 and LINC00475-S-induced mitochondrial fission in glioma cells, we performed RNA sequencing (RNA-seq). Overexpression of METTL3 resulted in 242 up-regulated genes and 198 down-regulated genes, which were enriched in various signaling pathways including apoptosis, lysosomes, mitophagy, RNA transport, and splicing (Fig. 5A and Fig. S4A). Inhibition of LINC00475-S led to 399 up-regulated genes and 138 down-regulated genes, which were also enriched in pathways of apoptosis, autophagy, tumors, and lysosomes (Fig. 5B and Fig. S4B). An intersection of 12 common regulatory genes was identified (Fig. 5C), including macrophage migration inhibitory factor (MIF) (Fig. 5D). These significantly expressed genes were enriched to spliceosome pathway by gene set enrichment analysis (GSEA) analysis (Fig. 5E). qRT-PCR demonstrated that both overexpression of METTL3 and LINC00475-S significantly reduced the expression of MIF, while inhibition of METTL3 and LINC00475-S markedly increased its expression (Fig. 5F and G). Overexpression of METTL3 could significantly down-regulate MIF proteins (Fig. 5H and Fig. S4C). The inhibition of LINC00475-S leads to a significant up-regulation of MIF expression, surpassing the effect observed with LINC00475 inhibition (Fig. 5I and Fig. S4D). Suppression of METTL3 remarkably increased MIF expression, while overexpression of LINC00475-S obviously down-regulated MIF proteins, whereas no effect was observed with LINC00475 in glioma cells. Surprisingly, MIF protein was barely detectable in U251 cells (Fig. 5H and I and Fig. S4C and D). To validate the precision of RNA-seq, we additionally assessed the function of METTL3 and LINC00475-S on the expression of APOE, PLIN2, and ISY1-RAB45. The results demonstrated that overexpression of METTL3 could reduce the mRNA levels of APOE and PLIN2 and enhance the levels of ISY1-RAB45; knockdown of LINC00475-S promoted the expression of APOE and PLIN2 and restrained the expression of ISY1-RAB45 in U251 cells (Fig. S4E). Overexpression of MIF restored mitochondrial fusion in U251 cells co-overexpressing with METTL3 (Fig. 5J) or LINC00475-S (Fig. 5K). In glioma cell cytoplasm, overexpression of MIF suppressed DRP1 and p-DRP1 expression while enhancing OPA1 and MFN2 expression. Co-overexpression of MIF and METTL3 partially restored DRP1 and p-DRP1 expression, but had no important effect on p-DRP1 levels in U87 cells. Additionally, it led to decreased OPA1 expression without notably affecting MFN2 levels (Fig. 5L and Fig. S4F). In the mitochondria of glioma cells, MIF overexpression markedly inhibited DRP1 and p-DRP1 expression while increasing OPA1 levels, with no substantial impact on MFN2 expression. Co-overexpression of MIF and METTL3 resulted in higher levels of DRP1 and p-DRP, as well as lower levels of OPA1 and MFN2 compared to the MIF-overexpressed group (Fig. 5L and Fig. S4E). The expression of DRP1 and p-DRP1 was reduced in the MIF and LINC00475-S co-expression group compared to the MIF-overexpressed group, along with increased expression of OPA1 and MFN2. This regulatory effect was particularly pronounced in mitochondria (Fig. 5M and Fig. S4G). These findings suggested that METTL3 and LINC00475-S promoted mitochondrial fission by suppressing MIF expression in glioma cells.

### HNRNPH1 participates in the AS of LINC00475 in glioma cells

**Fig. 6.**
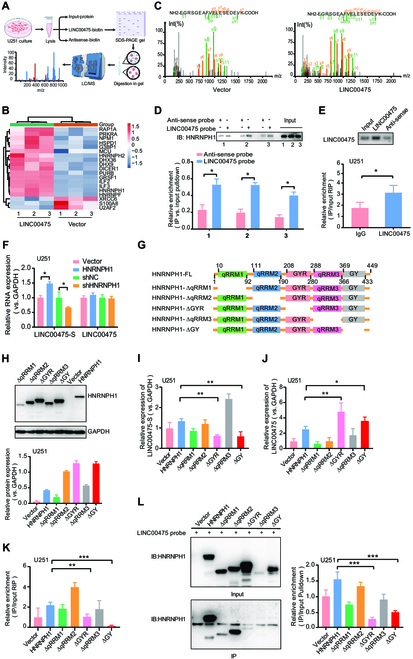
HNRNPH1 bound to LINC00475 in glioma cells. (A) A schematic illustration of RNA pulldown followed by mass spectrometry assay was presented to identify potential proteins interacting with the synthesized biotin-labeled LINC00475 probe. (B) Heatmap analysis revealed potential proteins pulled down by the synthesized biotin-labeled LINC00475 probe in U251 cells. (C) Mass spectrometry identified HNRNPH1 peptides pulled down by the synthesized biotin-labeled LINC00475 probe. (D) Western blotting confirmed the presence of HNRNPH1 protein pulled down by the synthesized biotin-labeled LINC00475 probe. (E) RIP assays demonstrated the enrichment capacity of HNRNPH1 to LINC00475 through anti-HNRNPH1 antibodies in U251 cells compared to IgG controls. (F) RT-qPCR analysis assessed relative expression levels of LINC00475 and LINC00475-S upon knockdown or overexpression of HNRNPH1 in U251 cells using shHNRNPH1/shNC/HNRNPH1/vector lentivirus, respectively. (G) Diagrams illustrating full-length or truncated versions of recombinant HNRNPH1 protein containing various assembled domains. (H) Full-length or truncated recombinant HNRNPH1 protein with correct size was validated by Western blotting using anti-HNRNPH1 in U251 cells. RT-qPCR was employed to assess the relative expression of LINC00475-S (I) and LINC00475 (J) in U251 cells infected with full-length or truncated recombinant HNRNPH1 lentivirus. (K) RT-qPCR detected the relative enrichment levels of LINC00475 in full-length or truncated HNRNPH1 RIP assays using anti-HNRNPH1 and anti-IgG in U251 cells. (L) Western blotting was utilized to identify the full-length or truncated recombinant HNRNPH1 protein pulled down by synthesized biotin-labeled LINC00475 probe. The measurement data were presented as mean ± SD. All tests in this study were repeated three times. *P < 0.05, **P < 0.01, ***P < 0.001.

To elucidate the mechanism by which METTL3 facilitates LINC00475 splicing in gliomas, we employed pulldown-LC/MS (liquid chromatography/mass spectrometry) to screen proteins that directly interact with LINC00475 (Fig. 6A). Nineteen significantly expressed proteins were identified (Fig. 6B), of which 16 exhibited increased binding affinity, while 3 showed decreased binding ability (Fig. S5A). Eukaryotic Orthologous Groups (KOG) analysis revealed that these proteins were involved in RNA processing, transcription, and posttranscriptional modification pathways (Fig. S5B). Gene ontology (GO) analysis confirmed their DNA or RNA binding capabilities (Fig. S5C). The interaction of m6A recognition protein HNRNPH1 with LINC00475 was enhanced through pulldown-LC/MS (Fig. 6C). Subsequently, pulldown-IP experiments confirmed a significantly strong binding capacity between the LINC00475 probe and HNRNPH1 (Fig. 6D). RIP assays further demonstrated a markedly higher binding between HNRNPH1 and LINC00475 (Fig. 6E). When HNRNPH1 was overexpressed, the expression of LINC00475-S was significantly up-regulated in U251 cells. Conversely, when HNRNPH1 was inhibited, the expression of LINC00475-S was markedly down-regulated; however, HNRNPH1 had no substantial impact on the LINC00475 expression (Fig. 6F and Fig. S5D). To identify the binding domain, we designed and constructed deletion mutants for each domain of HNRNPH1 (Fig. 6G) and confirmed their expression (Fig. 6H). Subsequently, qRT-PCR analysis revealed that deletion of GYR (glycine-tyrosine-arginine-rich) and GY (C-terminal glycine-rich) domains led to a significant decrease of LINC00475-S expression while significantly increasing the expression of LINC00475 (Fig. 6I and J). Furthermore, RIP experiments demonstrated a substantial reduction in interaction between HNRNPH1 and LINC00475 upon deletion of GYR and GY domain (Fig. 6K and Fig. S5E), which was further validated by pulldown-IP assays (Fig. 6L). These results indicated that the GYR and GY domain of HNRNPH1 interacts with LINC00475.

### METTL3 facilitates mitochondrial fission by inducing the interaction of HNRNPH1 and LINC00475 in glioma cells

**Fig. 7.**
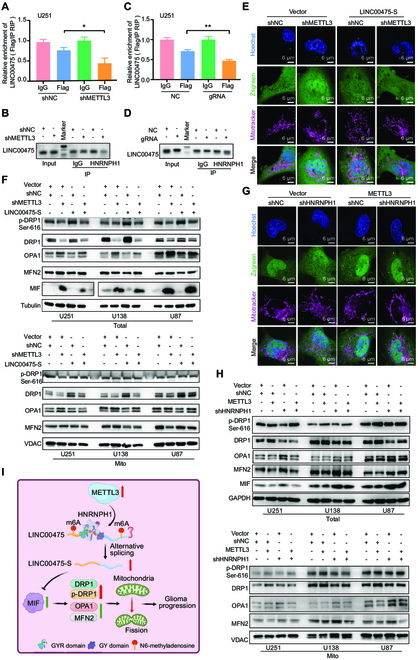
HNRNPH1 mediated METTL3/LINC00475 axis-induced mitochondrial fission. RIP assays were performed using anti-HNRNPH1 and anti-IgG antibodies in METTL3 knockdown U251 cells. The enrichments of LINC00475 by HNRNPH1 or IgG were quantified via RT-qPCR (A) and agarose gel images (B). RIP assays were performed using anti-HNRNPH1 and anti-IgG antibodies in demethylation-modified U251 cells. The enrichments of LINC00475 by HNRNPH1 or IgG were quantified via RT-qPCR (C) and agarose gel images (D). NC, negative control. (E) Confocal microscopy visualized the mitochondrial morphology in U251 cells transfected with METTL3 plus LINC00475-S knockdown plasmids, hoechst33254 for nuclear staining (blue), mitotracker for mitochondria (purple), zsgreen for vector (green). (F) The cellular (Total) and mitochondrial (Mito) protein levels of DRP1, p-DRP1, OPA1, and MFN2 were examined by Western blotting in METTL3-overexpressed and LINC00475-S knockdown samples. VDAC served as a positive control. (G) Confocal microscopy revealed the mitochondrial morphology in METTL3-overexpressed U251 cells infected with HNRNPH1 knockdown lentivirus, hoechst33254 for nuclear staining (blue), mitotracker for mitochondria (purple), zsgreen for vector (green). (H) Cellular and mitochondrial protein levels of DRP1, p-DRP1, OPA1, MFN2, and MIF in METTL3-overexpressed glioma cells were determined by Western blotting analysis following infection with HNRNPH1 knockdown lentivirus. GAPDH and VDAC were applied as positive controls. (I) The schematic diagram of mitochondrial fission induced by METTL3-promoted LINC00475 splicing was created by BioRender.com. The measurement data were presented as mean ± SD. All tests in this study were repeated three times. *P < 0.05.

Up-regulation of METTL3 stimulated cell proliferation and migration, while inhibition of METTL3 expression suppressed cell proliferation in glioma cells (Fig. S6A and B). Down-regulation of METTL3 significantly reduced the binding between HNRNPH1 and LINC00475 in U251 cells through RIP analysis (Fig. 7A and B and Fig. S6C). RIP detection demonstrated a decreased binding between LINC00475 and HNRNPH1 following demethylation modification (Fig. 7C and D and Fig. S6D). The inhibition of METTL3 in U251 cells significantly augmented mitochondrial fusion compared to the control group, whereas inhibition of METTL3 and co–up-regulated LINC00475-S partially rescued mitochondrial fission (Fig. 7E). Suppression of METTL3 profoundly decreased the expression of both DRP1 and p-DRP1, simultaneously up-regulating OPA1, MFN2, and MIF. However, the intervention of METTL3 with LINC00475-S overexpression partially rescued the expression of DRP1 and p-DRP1, as well as reduced the expression of OPA1, MFN2, and MIF in both whole cells and mitochondria (Fig. 7F and Fig. S6E). Mitotracker staining demonstrated that suppression of HNRNPH1 promoted mitochondrial fusion, and inhibition of HNRNPH1 in METTL3-overexpressed U251 cells impeded mitochondrial fusion (Fig. 7G). The knockdown of HNRNPH1 significantly decreased the expression of DRP1 and p-DRP1 but increased the levels of OPA1, MFN2, and MIF proteins in both whole cells and mitochondria. Moreover, overexpression of METTL3 combined with HNRNPH1 inhibition resulted in a slight decrease of DRP1 and p-DRP expression compared to the METTL3-overexpressed group, but an increase compared to only the HNRNPHI-inhibited group. Additionally, it led to elevated levels of OPA1, MFN2, and MIF compared to the METTL3-overexpressed group. However, these levels were significantly down-regulated compared to the HNRNPH1-inhibited group (Fig. 7H and Fig. S6F). In conclusion, METTL3-mediated m6A triggered mitochondrial fission by facilitating the binding between HNRNPH1 and LINC00475, thereby promoting glioma progression (Fig. 7I).

## Discussion

Limited research confirmed up-regulation of LINC00475 in glioma, and its inhibition could effectively suppress glioma growth both in vitro and in vivo [18,19]. However, the correlation analysis between LINC00475 and clinical parameters of glioma was not comprehensive, as it only focused on the association between LINC00475 expression and prognosis using TCGA database. In this study, we conducted a thorough investigation of the relationship between LINC00475 expression and recurrence, prognosis, age, pathological grade, IDH1 mutation status, as well as 1p/19q co-deletion, using both TCGA and CGGA databases. Our findings revealed inconsistent correlations between LINC00475 and clinical parameters of glioma across these two databases. This suggested that the clinical importance of detecting LINC00475 may vary among different populations. Notably, the expression of LINC00475 was significantly higher in IDH1 wild-type gliomas compared to IDH1 mutant gliomas. The accumulation of 2-hydroxyglutarate, mediated by IDH1, plays a crucial role in glioma progression, rendering IDH1 an important marker for molecular typing of glioma [20]. Most IDH1 wild-type GBMs survive for 15 to 18 months after diagnosis, with a 5-year survival rate not exceeding 10%. Conversely, IDH-mutant GBMs exhibit a better prognosis [21]. The findings of our research demonstrated a significant elevation of LINC00475 in IDH1 wild-type gliomas compared to IDH1-mutant gliomas, thereby corroborating a positive correlation between high LINC00475 levels and poor outcome in glioma patients. However, additional studies are required to explore the combined impact of IDH1 genotype and LINC00475 expression on glioma prognosis. The occurrence of 1p/19q co-deletion is predominantly observed in IDH1-mutant gliomas [22]. Moreover, proteomics has confirmed that 1p/19q co-deletion may not serve as a substantial marker for glioma progression [21]. Consequently, it can be reasonably inferred that LINC00475, being closely associated with glioma progression, does not exhibit a robust correlation with 1p/19q co-deletion.

In this study, the results suggest that AS of LINC00475 may take part in the development and recurrence of glioma. AS is a widespread transcriptional regulatory mechanism that enhances genomic coding capacity and promotes malignant tumor progression [23]. Tumors possess specific splice variants distinct from classical transcripts, which are often involved in processes such as apoptosis, DNA repair, proliferation, and migration during tumorigenesis [24]. Therefore, tumor-specific AS may be considered as a potential biomarker. In 2022, Su et al. [25] identified 9,028 AS events across 4,117 genes from TCGA database. This indicates that AS is a common phenomenon in gliomas; however, this research only focused on mRNA splicing while neglecting lncRNA splicing analysis. Due to the limitations of the online data, the capabilities were restricted to analyzing individual lncRNA rather than overall lncRNA splicing patterns in gliomas.

There is a lack of reports on the involvement of lncRNAs in regulating mitochondrial fission in gliomas. In recent years, research on lncRNAs involved in mitochondrial fission has primarily focused on myocardial injury, with only limited studies investigating their role in tumor cells. For instance, LL22NC03-N14H11.1 promotes hepatocellular carcinoma progression by promoting mitochondrial fission through mitogen-activated protein kinase (MAPK) pathway activation [26]. RACGAP1P accelerates breast cancer invasion and metastasis via miR-345-5p/RacGAP1 axis-mediated mitochondrial fission [27]. This study confirmed that LINC00475-S could promote mitochondrial fission in glioma cells by increasing DRP1 and p-DRP1 levels while down-regulating OPA1 and MFN2 expression.

Our study has verified the role of METTL3 in facilitating AS of LINC00475 and inducing m6A-dependent up-regulation of LINC00475-S. The regulatory function of METTL3 in lncRNAs AS in gliomas has not been previously documented. m6A modification regulates mRNA splicing, with enrichment observed in exons and introns during this process [28]. Knockdown of METTL3 can lead to AS changes in thousands of genes, thereby regulating AS in various tumor cells [29]. Inhibition of METTL3 expression in HepG2 cells results in differential exon and intron splicing patterns [30]. Additionally, METTL3 can regulate oncogenesis-related AS events in breast cancer, five of which are associated with poor prognosis [31]. METTL3 exhibits high expression in glioma and plays an oncogenic role. Suppression of METTL3 expression also leads to decreased m6A levels of serine- and arginine-rich splicing factor (SRSF), resulting in YTHDC1-dependent degradation of SRSF mRNA. This inhibition hampers the proliferation and self-renewal capacity of GBM stem cells [32]. However, this study did not investigate which AS events could be regulated by METTL3-mediated SRSF degradation. Maimaiti et al. [33] identified gene splicing associated with m6A modifiers as a potential prognostic marker for LGG patients through bioinformatics analysis. Nevertheless, experimental validation was lacking in their study. METTL3 sustains the stemness of glioma stem cells and confers radiotherapy resistance by promoting m6A methylation on LINC00839 [34]. It is evident that METTL3 can regulate both the expression and function of lncRNAs through m6A modification; however, its impact on lncRNA splicing has not been explored previously. Our study demonstrates that apart from mRNA regulation, METTL3 also governs lncRNA splicing.

In this study, we screened and identified HNRNPH1 as a facilitator for the regulation of LINC00475 m6A methylation by METTL3. HNRNPH1 is located on chromosome 5 and encodes a protein consisting of 449 amino acids. It contains three quasi-RNA recognition motifs (qRRMs), one GYR, and GY domains [35]. HNRNPH1 is verified to promote carcinogenesis. By activating the GRP78/p38 pathway, HNRNPH1 enhances the stability of LINC00662 and facilitates ovarian cancer progression [36]. HNRNPH1 regulates AS of target genes to promote tumor progression. Competitive binding between HNRNPH1 and SRSF3 to PRMT5 pre-mRNA promotes PRMT5-ISO5 production and increases radiosensitivity in hepatocellular carcinoma cells [37]. Pseudogene PRELID1P6 activates Akt/mammalian (or mechanistic) target of rapamycin (mTOR) pathway by increasing HNRNPH1-mediated TRF2 splicing and promoting glioma development [38]. The GYR and GY domains of HNRNPH1 are typical repetitive amino acid with low complexity domains (LCDs) that promote multivalent binding for regulation of multiple dynamic processes including gene expression [39]. Reversible phase separation of LCD is essential for interaction between HNRNPH1 and different classes of RNA-binding proteins as well as its ability to regulate AS [40]. Our study demonstrated that LCD was the key domain in HNRNPH1 regulating AS of LINC00475.

Recent studies have indicated the involvement of m6A modification in mitochondrial fission. METTL3 facilitates mitochondrial fission by augmenting the methylation of lncRNA GAS5 to induce myocardial fibrosis [41]. METTL3/14 inhibitor protects myocardial function by suppressing Drp1 m6A modification-mediated mitochondrial fission [42]. However, the impact of METTL3-mediated m6A modification on mitochondrial fission in tumor cells remains unexplored. This study validated that METTL3 promoted mitochondrial fission in glioma cells, thus fostering glioma progression.

MIF is a potent inflammatory marker capable of activating macrophages and T cells, thereby inducing inflammatory response. The role of MIF in tumor progression is multifaceted, as it can either inhibit or promote tumor growth through distinct signaling pathways [43]. Exosome-mediated transport of MIF has been shown to suppress temozolomide resistance in gliomas by modulating the TIMP3/phosphatidylinositol 3-kinase (PI3K)/AKT signaling axis [44]. High expression of MIF is associated with improved prognosis in GBM patients, and its expression significantly increases following neoadjuvant chemotherapy [45]. Furthermore, MIF can stimulate autophagy in GBM cells via activation of RhoA/ROCK1 pathway [46]. Studies have demonstrated that inhibition of MIF expression in tumor cells leads to down-regulation of OPA1 and MFN1 while up-regulating DRP1 expression, resulting in mitochondrial fragmentation [47]. The perplexing aspect of our findings was that despite METTL3 and LINC00475-S being identified as co-regulators of MIF through RNA-seq, MIF protein was absent in U251 cells but present in U138 and U87 cells. This suggested the possibility of posttranslational modification occurring specifically in U251 cells. Previous studies have demonstrated that phosphorylation, S-glycosylation, and S-nitrosation can take place at various sites on the MIF protein [48]. The investigation of posttranslational modifications in MIF in glioma requires further study.

Our study has some limitations. Since the online data did not provide information of lncRNA AS, we could not analyze the correlation between LINC00475-S expression and clinical parameters using bioinformatics methods. At the same time, we did not analyze the correlation between LINC00475 m6A levels and clinical parameters, so the importance of LINC00475 m6A clinical detection cannot be determined at present. In addition, we did not examine the percentage of spliced-in index values of LINC00475 in glioma samples. These will be further improved in our future work.

In summary, LINC00475-S exerted a stronger effect on promoting glioma progression by inducing mitochondrial fission compared to LINC00475. METTL3-dependent m6A methylation enhanced the binding of GYR and GY domains of HNRNPH1 to LINC00475, ultimately promoting glioma progression by inducing mitochondrial fission. Therefore, targeting AS events of LINC00475 may represent an effective strategy for inhibiting glioma progression.

## Materials and Methods

### Cell culture and transfection

Human glioma cell lines U87, U138, and U251 were purchased from Jennio (Guangzhou, China). All glioma cell lines were subjected to a short tandem repeat test. Cells were maintained in Dulbecco’s modified Eagle’s medium (DMEM) supplemented with 10% fetal bovine serum and 1% penicillin–streptomycin. We maintained cell lines at 37 °C in a 5% CO_2_ cell culture incubator and tested all cell lines routinely to exclude mycoplasma contamination.

Cell transfection was performed using Lipofectamine 3000 per the manufacturer’s instructions. Briefly, RNA oligonucleotides and the transfection reagent were separately diluted in Opti-MEM medium and incubated for 10 min at room temperature. Next, the two mixtures were combined and incubated for another 15 min at room temperature to allow the formation of transfection reagent–RNA complexes. The transfection complexes were then added to the cell culture medium dropwise. The cells were incubated for 24 h before changing to a fresh medium.

### Plasmid construction and lentiviral infection

For knockdown of LINC00475-S and LINC00475, specific small interfering RNAs (siRNAs) were synthesized from (RiboBio, Guangzhou, China). For the overexpression experiments, a full-length human LINC00475-S or LINC00475 sequence was obtained by PCR and subcloned into the pcDNA3.1(+) vector to establish cells that stably overexpressed LINC00475-S/LINC00475. Human METTL3 cDNA, MIF cDNA, HNRNPH1 cDNA, and its truncations were amplified from U251 cells and subcloned into pHBLV-CMV-MCS-3flag-EF1-ZsGreen-T2A-PURO vector. For METTL3/MIF/HNRNPH1 knockdown assay, two short hairpin RNAs (shRNAs) were designed using the guide design tool (https://www.sigmaaldrich.cn/) and were cloned into pHBLV-U6-MCS-CMV-ZsGreen-PGK-PURO. The lentivirus was collected 48 h after cotransfection of the lenti-gRNA, pCMV-vsvg, and pSPAX2 vector into HEK293T cells using Lipofectamine 3000 transfection reagent.

The pC0050-CMV-dPspCas13b-longlinker-ADAR2DD(wt) plasmids and pC0043-PspCas13b gRNA backbone plasmids were purchased from Addgene. Custom-designed guide RNAs (gRNAs) were cloned into pC0043-PspCas13b gRNA backbone, and the coding sequence (CDS) of ALKBH5 sequence was obtained by PCR. The construction of dPspCas13b-ALKBH5 vector was referred to the previous report [28]. ADAR2DD in pC0050-CMV-dPspCas13b-longlinker-ADAR2DD(wt) was replaced with ALKBH5. The sequences of related plasmids were listed in Table S1.

### Methylated RNA immunoprecipitation-qPCR

The primers were designed to amplify the high-m6A region (Table S1). Three hundred micrograms of extracted RNA was chemically fragmented into 150- to 250-nt fragments. Prewashed Pierce Protein A/G Magnetic Beads were incubated with 5 g of either rabbit immunoglobulin G (IgG) or anti-m6A antibody for 2 h at 4 °C while being rotated. After washing for three times, fragmented RNA and an immunoprecipitation solution containing ribonuclease (RNase) inhibitors were mixed with the antibody-attached beads overnight at 4 °C. Bound RNAs were incubated with 100 μl of elution buffer for 1 h at 4 °C, and then the methylated RNAs were precipitated using 5 mg of glycogen and 0.1 volume of 3 M sodium acetate in a 2.5 volume of 100% ethanol overnight at 80 °C. qPCR was used to quantify the additional enrichment, and after normalizing each sample to the input, the corresponding m6A enrichment was calculated.

### RNA immunoprecipitation

After 48 h, cells were transfected. About 1 × 10^7^ cells were pelleted and resuspended in 300 μl of lysis buffer [100 mM KCl, 5 mM MgCl_2_, 10 mM HEPES (pH7.0), 0.5% NP-40, 1 mM dithiothreitol, 100 U/ml RNase inhibitor]. Then, 200 μl of cell lysates was incubated with 5 μg of antibody against target protein or rabbit IgG-coated beads and rotated at 4 °C overnight. After treating the lysates with 100 μl of proteinase K buffer [50 mM tris–HCl (pH 7.4), 150 mM NaCl, 1 mM MgCl_2_, 0.05% NP-40, 30 μg of proteinase K], immunoprecipitated RNA was extracted by using RNAiso Plus. The extracted RNA was reverse transcribed to cDNA using the PrimeScript RT reagent Kit according to the manufacturer’s protocol. The abundance of LINC00475-S and LINC00475 was detected by qRT-PCR.

### RNA pull-down

To investigate the interaction between LINC00475 and HNRNPH1, the deletion domain protein was detected using an RNA pull-down assay kit. Briefly, 1 × 10^6^ U251 cells were collected and lysed. Probe-coated beads were generated by co-incubating the LINC00475 probe with Dynabead MyOne at room temperature for 1 h. The cell lysates were incubated with the LINC00475 probe at 4 °C for 1 h. After washing with wash buffer, the RNA complexes bound to the beads were eluted and extracted for spectrometry (MS) analysis or Western blot. The biotinylated LINC00475 probe was designed and synthesized by Gzscbio (Guangzhou, China).

### Design of gRNAs

According to the previous description [28], gRNAs were designed using http://chopchop.cbu.uib.no/. Given the presence of multiple transcripts for the target gene, an alignment analysis was performed on all transcript sequences. The common regions identified from this analysis were then utilized as potential targets for gRNA design. Specifically, gRNAs targeting CDS regions of the target transcripts were designed and listed in Table S1. Additionally, a U6 promoter-driven gRNA transcription system was cloned into the pC0043-PspCas13b gRNA backbone. To ensure specificity, all designed gRNAs underwent MEGABLAST (https://blast.ncbi.nlm.nih.gov/Blast.cgi) to avoid any mismatches with unexpected mRNAs in the human genome.

### Western blotting

Total proteins were extracted from treated human glioma cells using radioimmunoprecipitation assay (RIPA) buffer supplemented with a protease inhibitor cocktail. An equal amount of protein samples was then subjected to 10% or 12% sodium dodecyl sulfate–polyacrylamide gel electrophoresis (SDS-PAGE) and subsequently transferred onto polyvinylidene fluoride membranes through electrophoresis. The polyvinylidene difluoride (PVDF) membranes were blocked with 5% non-fat milk in tris-buffered saline containing 0.1% Tween 20 (TBST) for 1 h at room temperature, followed by overnight incubation with primary antibodies at 4 °C. After that, the membranes were incubated with either horseradish peroxidase (HRP)-conjugated secondary anti-mouse antibody or HRP-conjugated secondary anti-rabbit antibody at room temperature for 1 h. Finally, protein signals were detected using a chemiluminescence detection kit. Table S2 provides information on the antibodies used for METTL3, MIF, HNRNPH1, DRP1, p-DRP1-Ser616, OPA1 and MFN2, VDAC, tubulin, and GAPDH.

### SELECT qPCR

SELECT qPCR was performed according to Li and colleagues’ protocol [28]. Briefly, 1,500 ng of total RNA was mixed with 40 nM up primer, 40 nM down primer, and 5 μM deoxynucleotide triphosphate in a 17-μl solution containing 1× CutSmart buffer (NEB). The RNA and primers were subjected to a temperature gradient incubation: 90 °C for 1 min, 80 °C for 1 min, 70 °C for 1 min, 60 °C for 1 min, 50 °C for 1 min, and 40 °C for 6 min. Subsequently, the RNA and primer mixture was incubated with 3 μl of Bst2.0 DNA polymerase (0.01 U), SplintR ligase (0.5 U), and 10 nM ATP at 40 °C for 20 min, followed by denaturation at 80 °C for 20 min. Afterward, a qPCR volume of 20 μl was prepared containing 2 μl of the final reaction mixture and 200 nM SELECT primers in TB Green Premix Ex Taq II. SELECT qPCR was performed using the following program: 95 °C for 5 min; 95 °C for 10 s followed by 60 °C for 35 s repeated 40 cycles; 95 °C for 15 s and 60 °C for 1 min; 95 °C for 15 s and 4 °C hold. Primers used in SELECT qPCR or qRT-PCR are listed in Table S2. Ct values of samples were normalized to their corresponding Ct values of control. All assays were performed with three independent experiments.

### RNA sequencing

Total RNA was extracted from U251 cells (siLINC00475-S versus siNC or METTL3 versus vector) using RNAiso Plus following the manufacturer’s protocol. Subsequently, 5 μg of RNA underwent RNA-seq and the quality was evaluated using Agilent 2100 Bioanalyzer. High-throughput RNA-seq was performed on an Illumina HiSeq X Ten Sequencing System. The differentially expressed genes (DEGs) were filtered based on a significant threshold value (*P* < 0.05) and fold change (log_2_|FC| ≥ 1).

### Isolation of mitochondria

The mitochondria were isolated from cells grown in monolayer using a mitochondria isolation kit, following the manufacturer’s protocol. Briefly, after 48 h of cell transfection, the cell pellet was resuspended in lysis buffer and incubated on ice for 10 min. After centrifugation at 1,000*g* for 10 min at 4 °C, the resulting resuspension (containing cytoplasm) was collected for RNA or protein extraction, while the pellet was resuspended in disruption buffer. Complete cell disruption was achieved by slowly drawing the lysate into a syringe with a blunt-ended needle and ejecting it with one stroke 10 times. The lysate was then centrifuged at 1,000*g* for 10 min at 4 °C. The supernatant was carefully transferred to a clean 1.5-ml tube and further centrifuged at 6,000*g* for 10 min at 4 °C. The pellet was washed with storage buffer and subjected to another round of centrifugation at 6,000*g* for 20 min at 4 °C. Finally, the supernatant was carefully removed and the pellet was used for RNA or protein extraction.

### In vivo model

The animal studies were conducted with the approval of the Guangzhou Women and Children’s Medical Center (no. G2022-115). To establish in vivo tumors, 2.5 × 10^6^ U251 cells were subcutaneously implanted into the upper-right flank of nonobese diabetic (NOD)/severe combined immunodeficient (SCID) mice. After tumor formation at 4 to 6 weeks, the mice were randomly divided into three groups. Lentiviruses (LINC00475-S, LINC00475, and control vector) were packaged by OBiO (China) with a virus titer of 10^8^/units of infection (UI). A local injection of 5 × 10^6^/UI of virus was administered once every 2 weeks to the tumor mass. Tumor volume (longest diameter *A* and shortest diameter *B*) was measured every 7 d for 6 weeks. Upon reaching a tumor volume of 2 cm^3^, euthanasia was performed on the mice and tumor volume was calculated using the following formula: tumor volume (mm^3^) = (longest diameter × shortest diameter^2^)/2.

### Statistical analysis

Experiments were performed independently at least three times. Data were expressed as mean ± SD. Student’s *t* test or one-way analysis of variance (ANOVA) was used to assess statistical differences between two or multiple groups, respectively. The experimental data were analyzed using GraphPad Prism 9.0 software (GraphPad Inc., La Jolla, CA, USA). The Kaplan–Meier method was used to compute survival curves, which were analyzed by the log-rank test (*P* < 0.05).

## Data Availability

RNA-seq data reported in this paper have been deposited in the Genome Sequence Archive (Genomics, Proteomics & Bioinformatics 2021) in National Genomics Data Center (Nucleic Acids Res 2022), China National Center for Bioinformation/Beijing Institute of Genomics, Chinese Academy of Sciences (GSA-Human: HRA004880), which are publicly accessible at https://ngdc.cncb.ac.cn/gsa-human. The protein profile data reported in this paper have been deposited in the OMIX, China National Center for Bioinformation/Beijing Institute of Genomics, Chinese Academy of Sciences (https://ngdc.cncb.ac.cn/omix: accession no.OMIX004463). All remaining data are presented within the article and the Supplementary Materials, and available from the corresponding author upon request.
