# Supplementary material for: METTL3-Mediated LINC00475 Alternative Splicing Promotes Glioma Progression by Inducing Mitochondrial Fission
Source: Research (Wash D C). 2024 Feb 23;7:0324. doi: 10.34133/research.0324 (PMC10886067; doi:10.34133/research.0324)
Supplement: Supplementary 1 — Supplementary Materials and Methods Figs. S1 to S6 Tables S1 and S2 [file research.0324.f1.zip › Additional file 1-revised.docx]

**Supplementary materials and methods**

**Databases**

We obtained transcriptional and clinical data from the CGGA official website (http://www.cgga.org.cn/), and the TCGA database (https://tcga-data.nci.nih.gov/tcga/tcgaDownload.jsp). The splicing events of LINC00475 in glioma from TCGA database were analyzed using TSVdb database (http;//tsvdb.com). The m6A modification sites of LINC00475 were predicted using the SRAMP database ([http://www.cuilab.cn/sramp/)](http://www.cuilab.cn/sramp/)%5b27).

**Nucleoplasmic separation**

The cytoplasmic and nuclear fractions of glioma cells were isolated using a nuclear and cytoplasmic extraction kit, following the manufacturer's instructions. Subsequently, RNA samples were reverse-transcribed into cDNA and quantified using an RT-qPCR assay. GAPDH was used as a control for the cytoplasmic fraction, while U6 served as a control for the nuclear fraction. The 2^−ΔΔCt^method was employed to analyze the relative expression levels of genes in both compartments.

**Nucleic acid electrophoresis**

The cDNA and gDNA PCR products were analyzed using 1.5% agarose gel electrophoresis with TAE running buffer. Electrophoresis was performed at 110 V for 30 minutes to separate the DNA fragments, while DL1000 served as the DNA marker. The bands were visualized under UV irradiation.

**Realtime quantitative PCR**

Total RNA was extracted from cultured cells using the RNAiso Plus kit, following the manufacturer's protocol. The concentration and purity of RNA were determined spectrophotometrically using a multifunctional enzyme marker. cDNA of mRNA was synthesized using the PrimeScript^TM^ RT reagent Kit. Real-time quantitative PCR (RT-qPCR) assay was performed to evaluate gene expression levels using the TB Green^®^ Premix Ex Taq^TM^ II, according to the manufacturer's instructions. GAPDH was used as an endogenous control for both lncRNA and mRNA analysis, employing the 2^-ΔΔCt^ method. Each sample was run in triplicate. All primer sequences can be found in Supplementary Table S1.

**Cell proliferation assay (CCK-8)**

The cell proliferation was assessed using the Cell Counting Assay Kit-8 (CCK-8) following the manufacturer's instructions. Briefly, cells were transfected with plasmid or infected with lentivirus. Each group had at least three replicate wells. After 24 h, 48 h, 72 h, and 96 h, cells were harvested and incubated with ten microliters of CCK-8 solution for 1 hour. The absorbance at 450 nm was measured using an automatic multiwall spectrophotometer. Experiments were conducted at least three times with representative data.

**Transwell migration assay**

Fifty thousand transfected cells were suspended in 200 μl of FBS-free medium and plated in the upper chamber of each transwell assay insert. Subsequently, 600 μl of medium containing 10% FBS was added to the lower chamber. After incubation for 48 hours, the cells on the filter surface were fixed, stained with 0.5% crystal violet, and the number of invasive cells was counted under an inverted microscope.

**Immunofluorescent staining and fluorescence in situ hybridization**

The biotin-labeled probe for LINC00475-S and the digoxigenin-labeled probe for LINC00475 were synthesized by Biosense (Guangzhou, China) and are listed in Supplementary Table S1. Immunofluorescence staining was used to detect expression of METTL3 protein in the tissue. The tissue microarray (TMA), purchased from Alenabio Inc., contained a panel of 11 normal brain tissues, 35 low-grade gliomas (LGGs, Grade I-II), 17 high-grade gliomas (HGGs, Grade III), and 18 glioblastomas (GBMs, Grade IV). To determine the location of LINC00475-S and LINC00475, we used an lncRNA FISH kit (Exonbio, Guangzhou, China) according to the manufacturer's protocol. Briefly, after dewaxing in xylene and rehydration with ethanol at various concentrations followed by high-temperature repair in fresh citrate buffers for 10 minutes, tissue sections were hybridized with probes (1:100) in hybridization buffer at 37°C for 24 hours. After removing the probe and washing twice with PBS solution followed by blocking with 3% BSA for 30 minutes at 37°C, specific primary antibody was added along with rhodamine-conjugated anti-Digoxin and FITC-conjugated anti-streptavidin antibodies at a temperature of 4°C. After removing primary antibody cells were washed three times with PBS solution before incubating them with corresponding secondary antibody for another thirty minutes at a temperature of 37°C . Finally cell nuclei were counterstained using DAPI dye for fifteen minutes before obtaining images on Leica TCS SP8 confocal microscope. Each experiment was performed thrice.

**Electron microscopy**

The U251 cells were collected and immediately fixed in 2.5% glutaraldehyde for 24 hours, followed by fixation in 1% osmic acid for 1 to 2 hours. Subsequently, the cells were dehydrated with acetone and embedded in araldite CY212. The ultrathin sections were stained with alcoholic uranyl acetate and alkaline lead citrate, gently washed with distilled water, and observed using a JEM 1230 transmission electron microscope.

**Autophagic flux assays**

For autophagic flux assays, the mRFP-GFP-LC3 lentivirus was procured from HANBIO (Shanghai, China). U251 cells infected with the mRFP-GFP-LC3 lentivirus were transfected with 2 μg of plasmids in serum-free medium after 24 hours. Following a 48-hour incubation period, the cells were fixed using 4% paraformaldehyde and subsequently stained with Hoechst for nuclear visualization under confocal laser microscopy. Autophagy was assessed by quantifying the percentage of LC3-positive cells, with a minimum count of 5 cells per group.

**Liquid chromatography-tandem mass** **spectrometry**

The RNA pull-down production was prepared for LC-MS/MS analysis. The pull-down production was mixed with a solution containing 8M Urea and 100mM Tris-Cl, followed by sonication in a water bath. After centrifugation at 12000×g for 15 minutes, the supernatant was utilized for reduction reaction using 10mM DTT at 37 °C for 1 hour, followed by alkylation reaction with 40 mM iodoacetamide at room temperature in a dark place for 30 minutes. Protein concentration was determined using the Bradford method. The concentration of urea was reduced to below 2 M by diluting it with a solution containing 100 mM Tris-HCl (pH 8.0). Trypsin was added at a ratio of 1:50 (enzyme:protein, w/w) and allowed to digest overnight at 37 °C. The next day, TFA was used to adjust the pH to 6.0 and terminate the digestion process. After centrifugation at 12,000×g for 15minutes, the supernatant underwent peptide purification using Sep-Pak C18 desalting column.The eluted peptides were vacuum dried and stored at -20°C until further use.

The LC-MS/MS data acquisition was performed using a Q Exactive plus mass spectrometer coupled with an Easy-nLC 1200 system. A total of 10 μg peptides were loaded through an auto-sampler and separated on a C18 analytical column (50μm × 15cm, C18, 2μm, 100Å). To establish the separation gradient, mobile phase A (0.1% formic acid) and mobile phase B (80% ACN, 0.1% formic acid) were utilized. The separation was carried out at a constant flow rate of 300 nL/min. For DDA mode analysis, each scan cycle consisted of one full-scan mass spectrum (R = 70 K, AGC = 3e6, max IT = 20 ms, scan range = 350 – 1800 m/z), followed by 15 MS/MS events (R = 17.5 K, AGC = 2e5, max IT = 50 ms). The HCD collision energy was set to be at 28. An isolation window for precursor selection was set to be at 1.6 Da. Former target ion exclusion lasted for 35s.

The MS raw data were analyzed using MaxQuant (V1.6.6) and the Andromeda database search algorithm, with spectra files searched against the UniProt Human proteome database under the following parameters: LFQ mode for quantification was enabled, variable modifications included Oxidation (M), Acetyl (Protein N-term), and Deamidation (NQ), fixed modifications included Carbamidomethyl (C), digestion was performed using Trypsin/P, and a 20 ppm tolerance was set for MS1 matching.

**Flow cytometric analysis**

The Cell Apoptosis Analysis Kit was utilized for flow cytometric analysis. Briefly, the cells were detached using EDTA‐free trypsin and washed twice with PBS via centrifugation at 300 × g for 10 min at 4 °C. Subsequently, the cells were adjusted to a concentration of 1 × 10^6^ cells/ml using 250 μl of a 1 × Binding Buffer solution. Afterwards, 5 μl of Annexin V/PI was added to the cells, followed by incubation at room temperature in the dark for 10 min. Within an hour, the cells were transferred onto a flow cytometer. The apoptotic rate was calculated as the percentage of early plus late apoptotic cells using FlowJo v10.6.2 (FlowJo VX).

**Supplementary figure legend**

**Figure S1 The expression and splicing variant of LINC00475 in glioma tissues.**

A: The expression of LINC00475 in normal, primary and recurrent gliomas of GBM and LGG examined based on TCGA databases. B: The expression of LINC00475 in primary and recurrent gliomas with different WHO grades was analyzed using CGGA databases. The expression of LINC00475 in patients stratified by gender (C), age (D), IDH mutation status (E), 1p/19q co-deletion status (F) with different WHO grades was also investigated using CGGA databases. G: Splicing variants analysis of LINC00475 in LGG was predicted by TSVdb databases. H: RNA-FISH assays demonstrated the expression of LINC00475 and LINC00475-S in glioma tissue microarrays, nucleus was stained with DAPI (blue). I: RNA-FISH assays revealed the expression of both LINC00475 and LINC00475-S in glioma tissue microarrays including normal tissues, GBM and LGG, nucleus were stained with DAPI (blue).

**Figure S2 LINC00475-S induced autophagy in glioma cells.**

A: RT-qPCR detected the expression levels of LINC00475 and LINC00475-S upon overexpression or siRNA-mediated knockdown in U251 and U87 cells. B: Flow cytometry measurements assessed apoptotic rates after transfection with either overexpressing plasmids or siRNA targeting for both LINC00475 and LINC00475-S in U251 and U87 cells. C: An image-based co-localization analysis of mRFP-GFP-LC3 was used to evaluate autophagy flux following overexpressing plasmids or siRNA-mediated knockdown of both LINC00475 and LINC00475-S in U251 cells, nucleus were stained with DAPI (blue), GFP (green), mRFP (red). D: Western blotting assays showed protein levels of LC3 II/I and p62 after overexpression or knockdown of LINC00475 and LINC00475-S in U251 and U87 cells. GAPDH was used as internal control. The measurement data were presented as mean ± SD. All tests in this study were repeated three times. *P < 0.05, ** P < 0.01, ***P < 0.001, NS, non-significant.

**Figure S3 The expression of METTL3 was related with LINC00475-S and induced mitochondrial fission in glioma cells.**

A: Western blotting assays were performed to assess the protein levels of METTL3, ALKBH5, and FTO in glioma cells. B: Western blotting assays were conducted to examine the protein levels of METTL3 in U251 cells infected with METTL3 overexpressing lentivirus or shRNA. C: Western blotting assays were carried out to determine the protein levels of FTO and ALKBH5 in U251 cells infected with FTO and ALKBH5 overexpressing lentivirus. D: RT-qPCR assays demonstrated the relative expression of LINC00475-S and LINC00475 in U251 cells infected with ALKBH5 or FTO overexpressing lentivirus. E: Statistical analysis of FISH assay which detected the presence of METTL3 in normal brain tissues and glioma samples from tissue microarrays. F: The fluorescence intensity of METTL3 in GBMs and LGGs of tissue microarrays. The correlation between METTL3 mRNA, LINC00475-S (G) and LINC00475 in LGGs (H) tissues was measured using Pearson’s Correlation analysis. Pearson's Correlation analysis was used to measure the correlation between METTL3 mRNA expression LINC00475-S (I) and LINC00475 (J) in GBMs. Statistical diagram of Figure 3F (K), Figure 3H (L), Figure 3J (M) and Figure 3K (N). The measurement data were presented as mean ± SD. All tests in this study were repeated three times. *P < 0.05, ** P < 0.01, ***P < 0.001.

**Figure S4 MIF was involved in METTL3 and LINC00475-mediated mitochondrial fission in glioma cells.**

KEGG analysis using RNA-seq data after METTL3 knockdown (A) or LINC00475 overexpression (B) in U251 cells. Statistical diagram of Figure 4H (C) and Figure 4I (D). E: RT-qPCR detected the expression levels of APOE, PLIN2 and ISY1−RAB43 after overexpression of METTL3 or knockdown of LINC00475 in U251 and U87 cells. Statistical diagram of Figure 4L (F) and Figure 4M (G). The measurement data were presented as mean ± SD. All tests in this study were repeated three times. *P < 0.05.

**Figure S5 Proteins interacted with LINC00475-S in glioma cells.**

A: The volcano plot was utilized to analyze the binding proteins pulled down by the synthesized biotin-labeled LINC00475 probe in U251 cells. Red dots represent upregulated proteins, blue dots represent downregulated proteins, and gray dots represent proteins that were not differentially expressed (P < 0.05, |log2FC|≥0.5). The KOG (B) and GO (C) were employed to analyze the binding proteins pulled down by the synthesized biotin-labeled LINC00475 probe. D: Western blotting analysis was conducted to examine HNRNPH1 expression in U251 cells infected with HNRNPH1 overexpressing or shRNA lentivirus, GAPDH serving as internal control. E: RIP-PCR assays and agarose gel images were performed using an anti-HNRNPH1 antibody in U251 cells overexpressing different segments of HNRNPH1 constructs. The measurement data were presented as mean ± SD. All tests in this study were repeated three times. ** P < 0.01, ***P < 0.001.

**Figure S6 METTL3 promoted glioma progression through regulating LINC00475-S**

A: CCK-8 assays were used to assess cell viability in U251 and U87 cells infected with METTL3 overexpressed or shRNA lentivirus. B: Transwell assays demonstrated the migration abilities of U251 and U87 cells infected with METTL3 overexpressed or shRNA lentivirus. Statistical diagram of Figure 6B (C), Figure 6D (D), Figure 6F (E) and Figure 6H (F). The measurement data were presented as mean ± SD. All tests in this study were repeated three times. *P < 0.05, ** P < 0.01, ***P < 0.001.
