# Supplementary figures and images for: METTL3-Mediated LINC00475 Alternative Splicing Promotes Glioma Progression by Inducing Mitochondrial Fission
Source: Research (Wash D C). 2024 Feb 23;7:0324. doi: 10.34133/research.0324 (PMC10886067; doi:10.34133/research.0324)

Figure S1

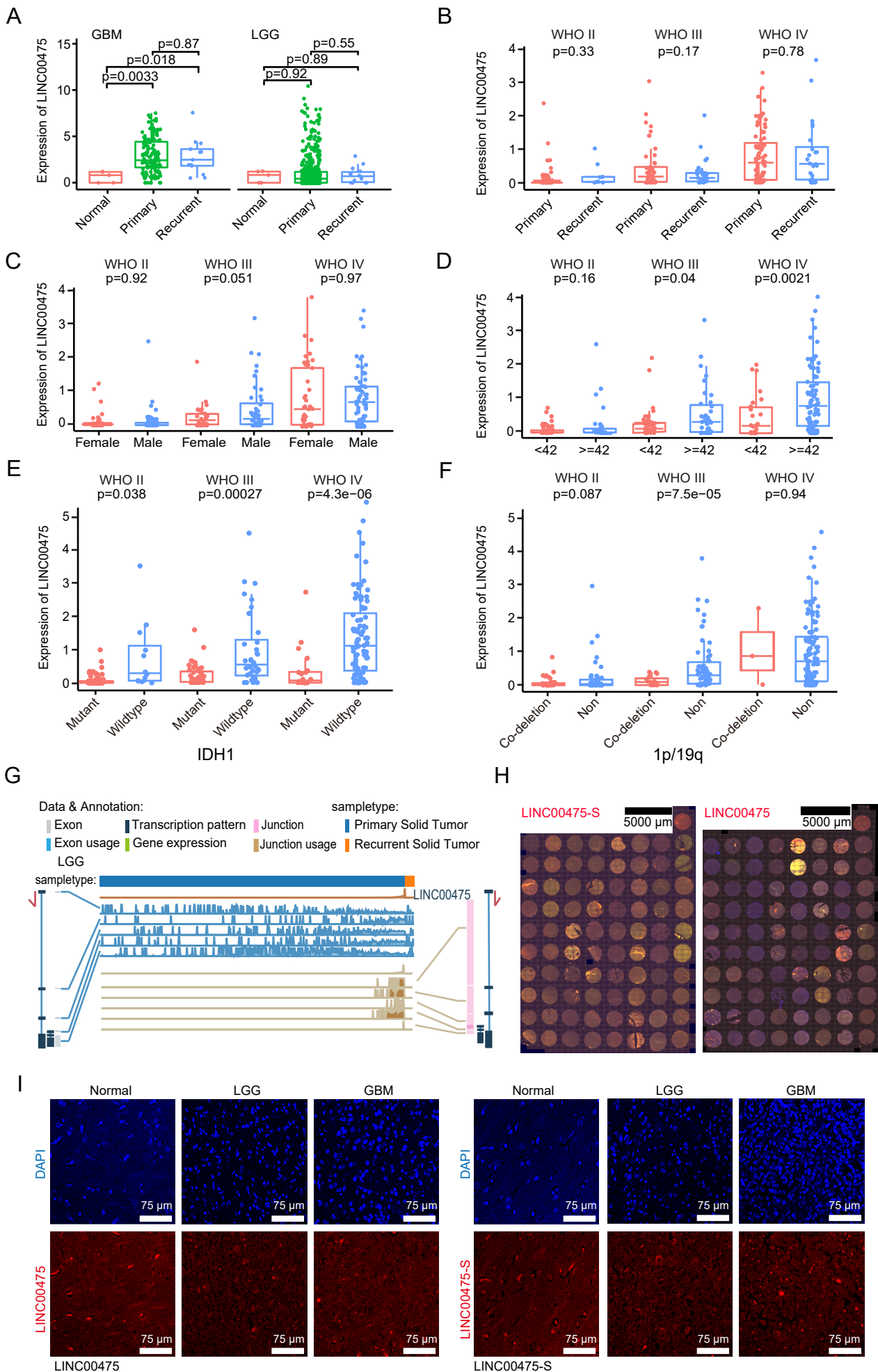

Supplement: Supplementary 1 — Supplementary Materials and Methods Figs. S1 to S6 Tables S1 and S2 [file research.0324.f1.zip › FigureS1.pdf]

Figure S2

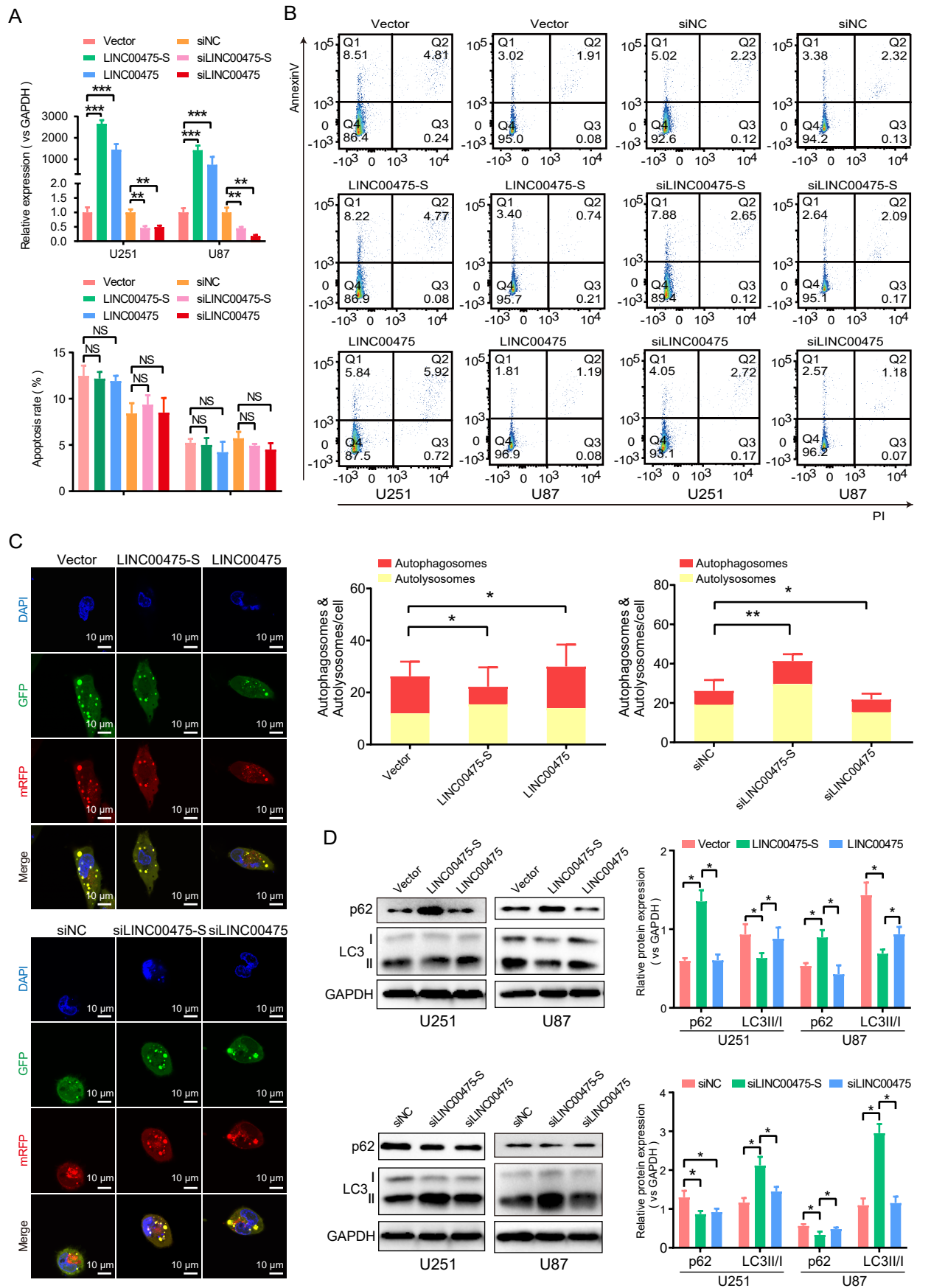

Supplement: Supplementary 1 — Supplementary Materials and Methods Figs. S1 to S6 Tables S1 and S2 [file research.0324.f1.zip › FigureS2.pdf]

Figure S3

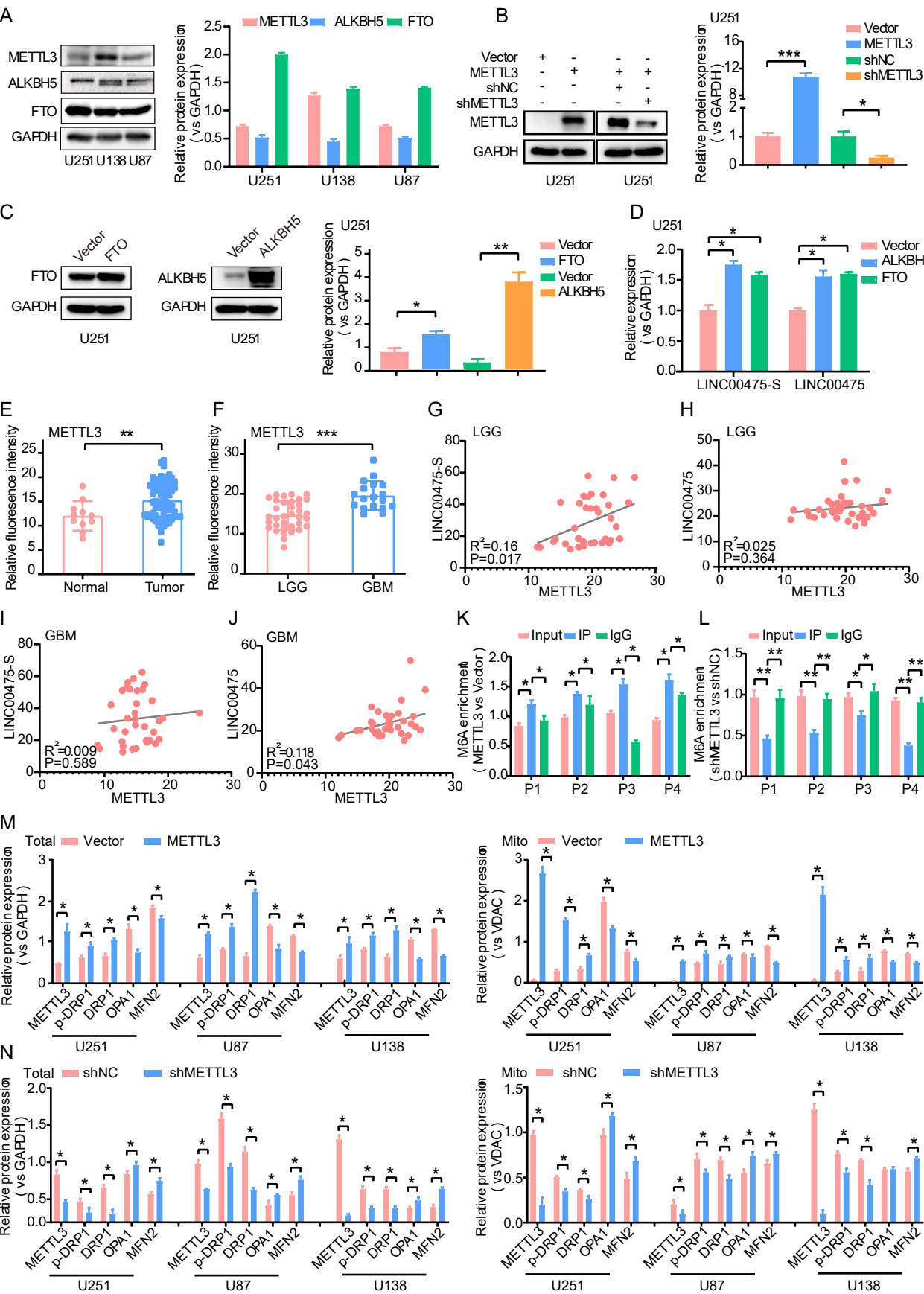

Supplement: Supplementary 1 — Supplementary Materials and Methods Figs. S1 to S6 Tables S1 and S2 [file research.0324.f1.zip › FigureS3.pdf]

Figure S4

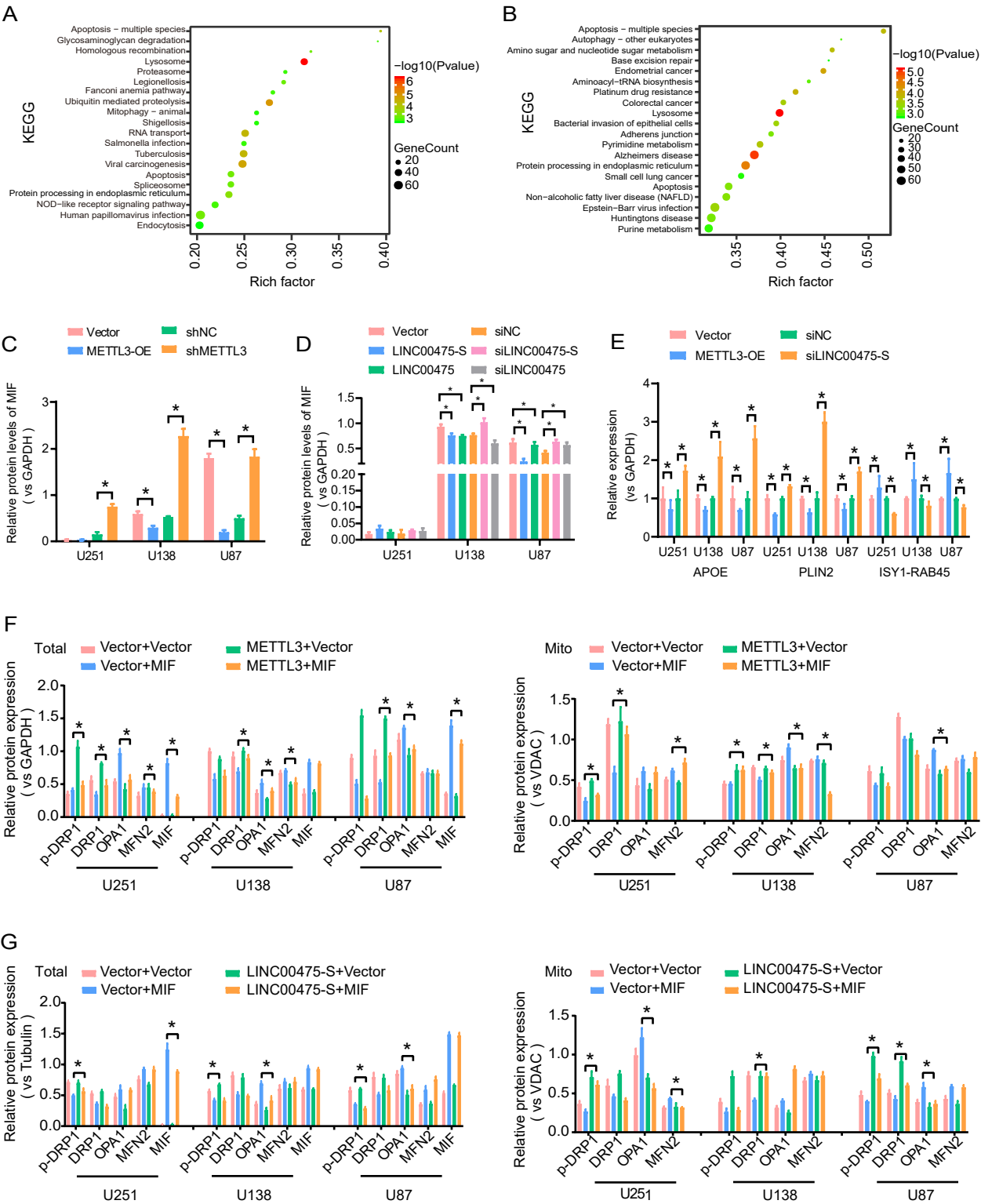

Supplement: Supplementary 1 — Supplementary Materials and Methods Figs. S1 to S6 Tables S1 and S2 [file research.0324.f1.zip › FigureS4.pdf]

Figure S5

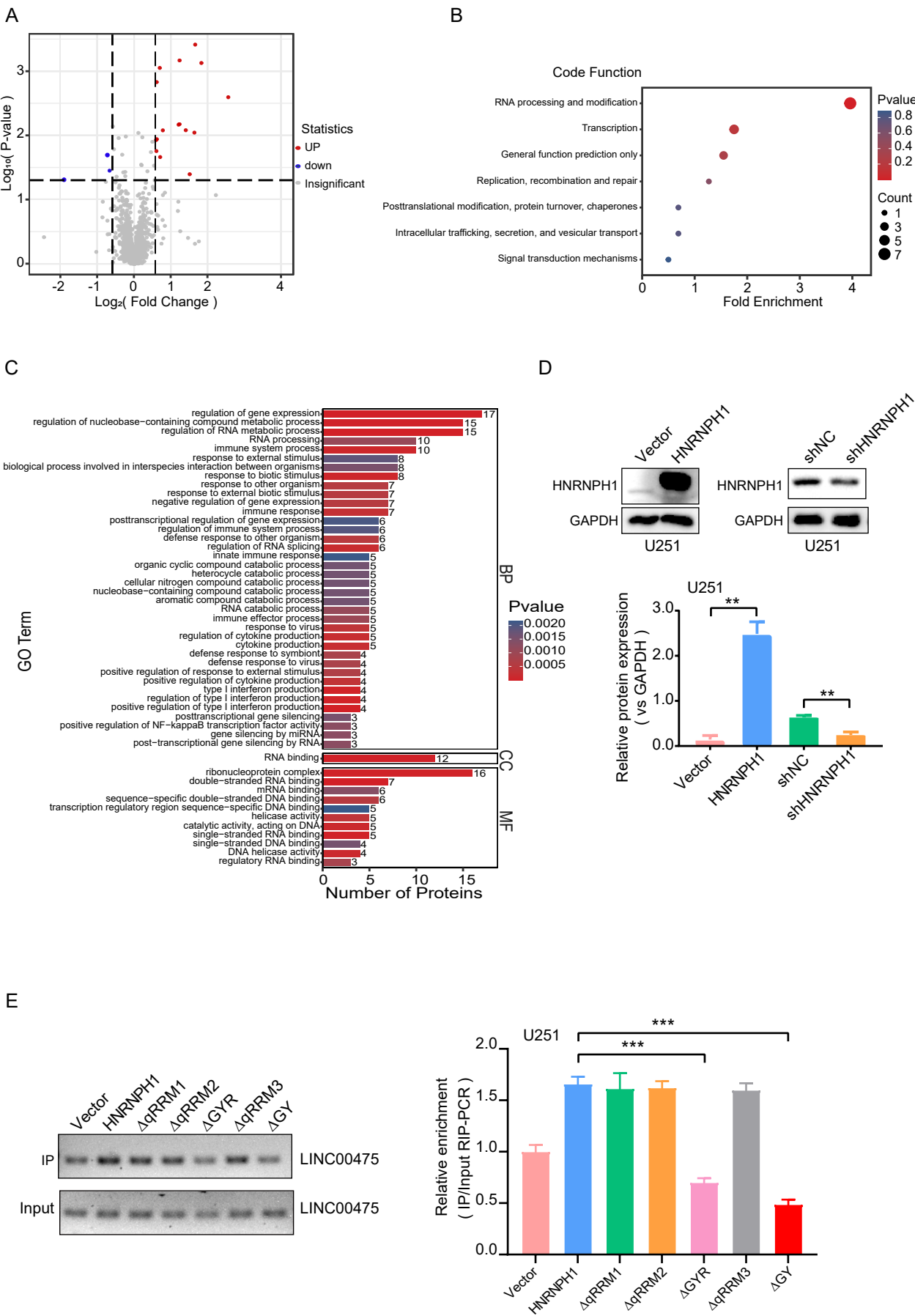

Supplement: Supplementary 1 — Supplementary Materials and Methods Figs. S1 to S6 Tables S1 and S2 [file research.0324.f1.zip › FigureS5.pdf]

Figure S6

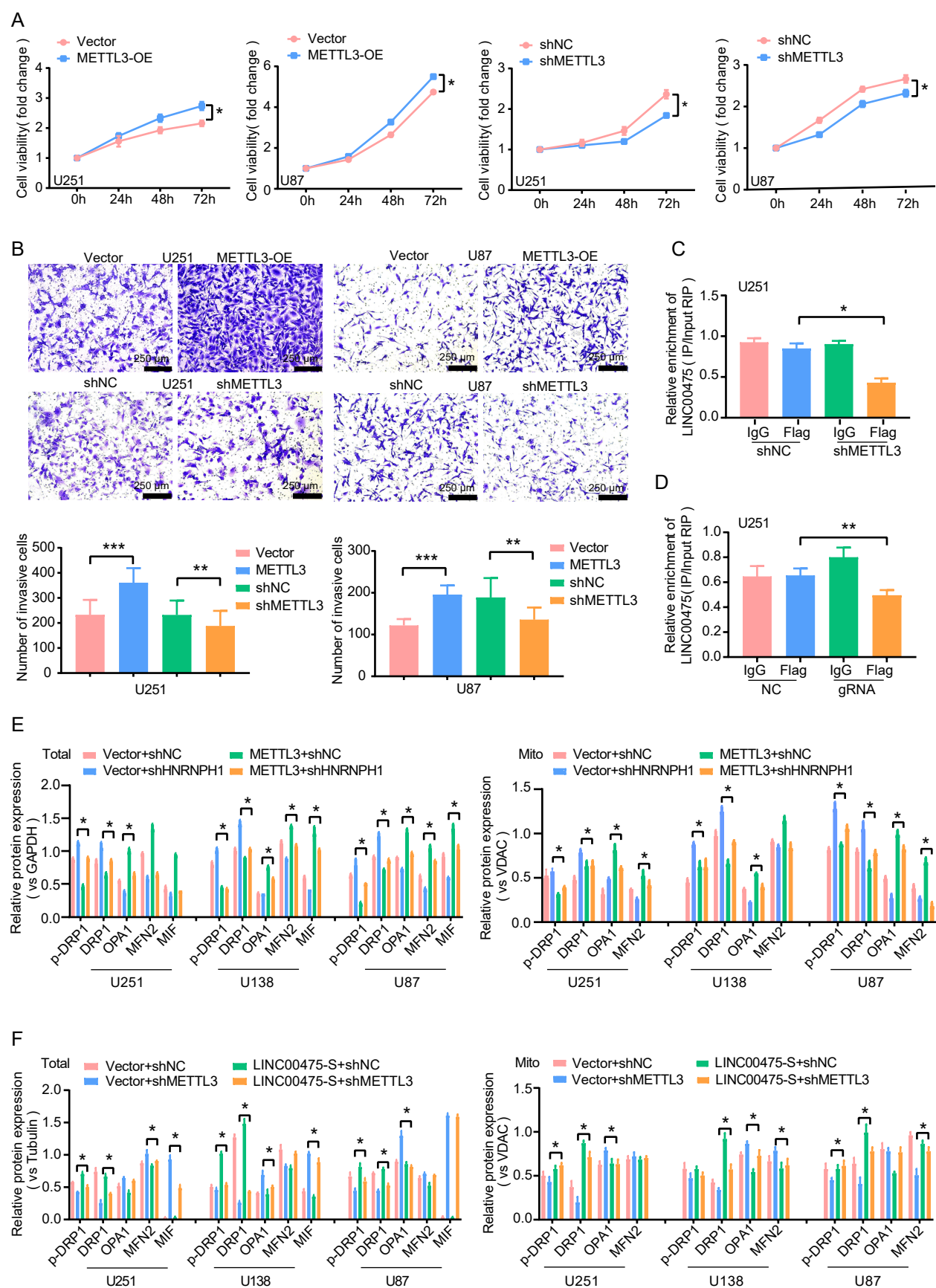

Supplement: Supplementary 1 — Supplementary Materials and Methods Figs. S1 to S6 Tables S1 and S2 [file research.0324.f1.zip › FigureS6.pdf]
